# Supplementary material for: A molecular mechanosensor for real-time visualization of appressorium membrane tension in Magnaporthe oryzae
Source: Nat Microbiol. 2023 Jul 20;8(8):1508–19. doi: 10.1038/s41564-023-01430-x (PMC10390335; doi:10.1038/s41564-023-01430-x)
Supplement: Supplementary file 1 — Reporting Summary [file 41564_2023_1430_MOESM1_ESM.pdf]

nature portfolio

Corresponding author(s): Nicholas J Talbot

Last updated by author(s): 2023/5/15

Reporting Summary

Nature Portfolio wishes to improve the reproducibility of the work that we publish. This form provides structure for consistency and transparency in reporting. For further information on Nature Portfolio policies, see our Editorial Policies and the Editorial Policy Checklist.

Statistics

For all statistical analyses, confirm that the following items are present in the figure legend, table legend, main text, or Methods section.

n/a	Confirmed	
☐	☒	The exact sample size (*n*) for each experimental group/condition, given as a discrete number and unit of measurement
☐	☒	A statement on whether measurements were taken from distinct samples or whether the same sample was measured repeatedly
☐	☒	The statistical test(s) used AND whether they are one- or two-sided<br>*Only common tests should be described solely by name; describe more complex techniques in the Methods section.*
☒	☐	A description of all covariates tested
☒	☐	A description of any assumptions or corrections, such as tests of normality and adjustment for multiple comparisons
☐	☒	A full description of the statistical parameters including central tendency (e.g. means) or other basic estimates (e.g. regression coefficient) AND variation (e.g. standard deviation) or associated estimates of uncertainty (e.g. confidence intervals)
☐	☒	For null hypothesis testing, the test statistic (e.g. *F*, *t*, *r*) with confidence intervals, effect sizes, degrees of freedom and *P* value noted<br>*Give P values as exact values whenever suitable.*
☒	☐	For Bayesian analysis, information on the choice of priors and Markov chain Monte Carlo settings
☒	☐	For hierarchical and complex designs, identification of the appropriate level for tests and full reporting of outcomes
☒	☐	Estimates of effect sizes (e.g. Cohen's *d*, Pearson's *r*), indicating how they were calculated

Our web collection on statistics for biologists contains articles on many of the points above.

Software and code

Policy information about availability of computer code

Data collection	A real time FLIM movie was captured for appressorium turgor generation between 4-7.5h over 146 min. FLIM imaging was performed on a Stellaris 8 Falcon upright scanning confocal microscope (Leica systems). Other FLIM imaging was performed on a LEICA TCS SP8X upright scanning confocal microscope coupled to a PicoHarp 300 TCSPC module (PicoQuant GmbH).
Data analysis	Stellaris images were processed in LAS X (version 4.2, Leica Microsystems). Movies were generated using Python (Anaconda Software Distribution. Computer software. Vers. 3.8.10). Anaconda.2016. LEICA TCS SP8X images were analysed using SymPhoTime 64 (version 2.4, PicoQuant GmbH). The code used to generate the movies for the FLIM time series can be found at https://github.com/SergioGabrielLopez/movie_script.

Data

Policy information about availability of data

All manuscripts must include a data availability statement. This statement should provide the following information, where applicable:

- Accession codes, unique identifiers, or web links for publicly available datasets
- A description of any restrictions on data availability
- For clinical datasets or third party data, please ensure that the statement adheres to our policy

All data that support the findings of this paper are available from the corresponding author on request.

Human research participants

Policy information about studies involving human research participants and Sex and Gender in Research.

Reporting on sex and gender	Not applicable
Population characteristics	Not applicable
Recruitment	Not applicable
Ethics oversight	Not applicable

Note that full information on the approval of the study protocol must also be provided in the manuscript.

Field-specific reporting

Please select the one below that is the best fit for your research. If you are not sure, read the appropriate sections before making your selection.

☒ Life sciences ☐ Behavioural & social sciences ☐ Ecological, evolutionary & environmental sciences

For a reference copy of the document with all sections, see nature.com/documents/nr-reporting-summary-flat.pdf

Life sciences study design

All studies must disclose on these points even when the disclosure is negative.

Sample size	Sample sizes were as large as practicable for appressorium FLIM capturing and analysis, based on previous studies where estimates have provided statistically significant findings. In all cases we used three biological replicates for experiments, as that is standard practice for our studies in our field to ensure reproducibility of the presented results (for examples: Ryder *et al.*, 2019 Nature 574, pages 423-427, Michels *et al.*, 2020 PNAS 117(30), pages 18110-18118.
Data exclusions	No data were excluded from any part of this study.
Replication	All experiments were subject to at least three biological replications unless otherwise stated.Technical replications were also carried out as stated in the text Results were consistent between replication unless otherwise stated.
Randomization	Microscopy observation and quantification were from samples selected randomly and quantified independently several times. Randomization in experimental procedures (such as membrane imaging for lifetime quantification) was not necessary because experiments were performed under well controlled conditions or treatments. No animal or humans specimens were used in this study and randomization is not generally used in this field.
Blinding	Blind testing was not routinely carried out in the study as it was not relevant to most of the experiments carried out. Blinding was not necessary because every experiment was quantified three independent times with several technical replicates.

Reporting for specific materials, systems and methods

We require information from authors about some types of materials, experimental systems and methods used in many studies. Here, indicate whether each material, system or method listed is relevant to your study. If you are not sure if a list item applies to your research, read the appropriate section before selecting a response.

Materials & experimental systems

n/a	Involved in the study
☒	☐ Antibodies
☒	☐ Eukaryotic cell lines
☒	☐ Palaeontology and archaeology
☒	☐ Animals and other organisms
☒	☐ Clinical data
☒	☐ Dual use research of concern

Methods

n/a	Involved in the study
☒	☐ ChIP-seq
☒	☐ Flow cytometry
☒	☐ MRI-based neuroimaging
